# Antimicrobial peptide-modified AIE visual composite wound dressing for promoting rapid healing of infected wounds

**DOI:** 10.3389/fbioe.2023.1338172

**Published:** 2024-01-12

**Authors:** Yi Chen, Hongjin Qian, Dandan Peng, Yan Jiang, Qiaolin Liu, Yan Tan, Longbao Feng, Biao Cheng, Guilan Li

**Affiliations:** ^1^ Department of Cadre Ward, General Hospital of Southern Theater Command, Guangzhou, China; ^2^ Department of Oncology, General Hospital of Southern Theater Command, Guangzhou, China; ^3^ Key Laboratory of Biomaterials of Guangdong Higher Education Institutes, Department of Biomedical Engineering, Guangdong Provincial Engineering and Technological Research Centre for Drug Carrier Development, Jinan University, Guangzhou, China; ^4^ Department of Burns and Plastic Surgery, General Hospital of Southern Theater Command, Guangzhou, China; ^5^ Department of Neurosurgery, General Hospital of Southern Theater Command, Guangzhou, China

**Keywords:** antimicrobial peptide, PVA-TPE, wound dressing, smart response, healing

## Abstract

Wound infection is a major problem faced during wound healing. Therefore, it is necessary to develop wound dressings with excellent antimicrobial properties. Here, a smart response system of PVA-TPE/HA-AMP/SF/ALG wound dressing was prepared by a combination of chemical cross-linking and freeze-drying methods. We grafted AMP onto HA to endow the wound dressing with bacterial resistance and slow release of AMP. At the same time, the system detects bacterial activity in real time for precise antimicrobial activity (through the use of PVA-TPE) and modulates inflammation to reduce bacterial infection (through the use of AMP). In addition, the PVA-TPE/HA-AMP/SF/ALG wound dressing has a good three-dimensional mesh structure, which promotes cell proliferation, enhances collagen deposition and angiogenesis, and thus effectively promotes rapid healing of infected wounds. Moreover, it can induce the expression of inflammatory factors such as VEGF, TNF-α, IFN-γ, IL-4 and TGF-β1 in infected wounds through the Wnt/CAMK/p-PKC signaling pathway, inhibit inflammatory responses, promote wound healing and reduce scar formation. Therefore, the PVA-TPE/HA-AMP/SF/ALG wound dressing smart response system shows great promise in infected wound healing.

## 1 Introduction

Wounds are most commonly referred to as the site of damaged and torn skin, muscle, mucous membranes, etc., in humans or other animals. Wound healing is a healing process after skin and other tissues are broken or damaged, aiming to maintain the functionality and integrity of damaged tissues (Gurtner et al., 2008; Tang et al., 2023). It is a multi-step process consisting of four overlapping phases: hemostasis, inflammatory response, proliferation and remodeling (Gurtner et al., 2008; Hao et al., 2020). Wound infection is the most serious interfering factor in the wound healing process, which is mainly manifested by the destruction of the remaining epithelial tissue and in severe cases leads not only to surgical failure but even to amputation of the patient’s limb (Dow et al., 1999; Bucknall, 2005). Therefore, the promotion of wound healing and the reduction of wound infection rates are the focus of current medical research (Chung et al., 2017; Zhao X. et al., 2018; Qu et al., 2019). To date, a large number of wound dressings have been developed to reduce inflammation, control wound infection, promote neovascularization and facilitate wound healing (Lumbreras-Aguayo et al., 2019; Montaser et al., 2020). Depending on their form, they can be classified as gauze (He et al., 2020), hydrogel (Liang et al., 2019), nano-microspheres (Sun et al., 2017) and sponges (Xie et al., 2021). Although a wide variety of wound dressings have been developed and used in clinical practice, existing dressings still fail to meet some requirements. For example, most current clinical dressings are not able to adapt their performance to changing wound conditions (Dong and Guo, 2021). At the same time, they are unable to monitor changes in factors that trigger chronic wounds, such as infection (Zhao R. et al., 2016; Withycombe et al., 2017) and hyperglycaemia (Yu et al., 2019). Therefore, the development of smart responsive wound dressings is important for wound healing.

Sodium alginate (ALG) is a naturally occurring polymeric polysaccharide derived mainly from brown algae and consists of the basic structural units *ß*-D-mannuronic acid (M-unit) and α-L-guluronic acid (G-unit) linked by glycosidic linkages (Varaprasad et al., 2020; Zhang and Zhao, 2020). Sodium alginate has a similar molecular structure to the human extracellular matrix (ECM) and can be fabricated into a three-dimensional scaffold material as a supportive matrix for wound repair (Venkatesan et al., 2015; Rajesh et al., 2017). In addition, it is widely used in biomedical fields such as slow-release drug delivery and tissue engineering due to its easy accessibility, safety, non-toxicity, high stability and plasticity (Varaprasad et al., 2020). Silk fibroin (SF) is a natural polymer extracted from silk, the secretion of the domestic silkworm (Vepari and Kaplan, 2007). It has been extensively studied for its excellent mechanical properties, good biocompatibility, controlled biodegradability and ease of processing (Park et al., 2018; Wang et al., 2021). In addition, filipin scaffolds with a large number of interconnected pore structures play an important role in cellular nutrient and metabolic exchange (Kim et al., 2005; Yan et al., 2019; Hu et al., 2020; Tang et al., 2020). Currently, it has been reported that ALG/SF composite scaffolds can promote cell proliferation, accelerate wound closure and reduce inflammatory responses (Wang et al., 2022). Meanwhile, ALG/SF composite scaffolds are promising as a matrix for smart-responsive wound dressings. Biomedical materials commonly used for wound repair have antimicrobial properties (Zahra et al., 2023). However, ALG/SF composite scaffolds lack certain antimicrobial properties that play a negative role in wound repair. Therefore, improving their antimicrobial properties is more helpful to develop their potential as effective wound dressings (Yin et al., 2022).

Antimicrobial peptide (AMP) has become a hot new antimicrobial drug with wide application prospects due to its advantages of broad antimicrobial spectrum, high activity, diversity and target strains that are not easy to produce drug resistant mutations (Williams and Bax, 2009). The most common antibacterial mechanism is that antimicrobial peptides target the cytoplasmic membrane, depolarize the lipid bilayer structure and increase membrane permeability, leading to efflux of bacterial contents, thus achieving a rapid antimicrobial effect that can be achieved within a few seconds of contact with microorganisms (Ma et al., 2016; Peng et al., 2019; Chen H. et al., 2023). However, AMP alone suffers from instability, susceptibility to degradation by protein hydrolases, toxicity and other adverse effects (Yang et al., 2019). Therefore, AMP is always combined with other polymeric wound dressing materials to prolong the antimicrobial activity of AMP and reduce its cytotoxicity (Annabi et al., 2017; Wei et al., 2021), as well as to improve the antimicrobial properties of wound dressings and accelerate wound healing (Lozeau et al., 2020; Yang et al., 2021). In addition, the development of smart-responsive wound dressings capable of monitoring bacterial infections is the focus of current research.

Aggregation-induced emission luminoge (AIEgen) has unique luminescent properties, i.e., it can significantly enhance the luminescence intensity in the aggregated state, which makes it promising for applications in the fields of bioimaging and bio-probe (Gao et al., 2016). Meanwhile, AIEgen also has the advantages of good solubility, biocompatibility, low cytotoxicity (Qi et al., 2019), and the ability to fluorescently monitor bacterial activity in real time and guide precise antimicrobial therapy (Ge et al., 2022), making it an ideal tool for wound healing research. Fluorescent probes prepared from AIEgen tetraphenylene vinyl (TPE) derivatives (Yu et al., 2021) can be used to detect bacterial infections. *Staphylococcus aureus* (*Staphylococcus aureus*) is one of the most common bacteria causing wound infections and this probe is highly sensitive and specific for it (Li et al., 2017). Polyvinyl alcohol (PVA) has unique advantages in the field of biomedical materials due to its excellent biocompatibility and adhesion properties (Massarelli et al., 2021). TPE with tertiary amine groups can be easily grafted onto the side hydroxyl groups of PVA to form a derivative (PVA-TPE) with real-time and sensitive bacterial detection capability, which is the basis for accurate treatment of bacterial infections (Wang et al., 2020).

Therefore, in the present study, we proposed several hypotheses. First, we grafted AMP onto HA, assuming that it could overcome bacterial resistance and unrestricted release of AMP. Second, a PVA-TPE/HA-AMP/SF/ALG wound dressing smart response system was prepared by chemical crosslinking (EDC/NHS), which was hypothesized to detect bacterial activity in real time, achieve precise antimicrobial activity (through the use of PVA-TPE), regulate inflammation, reduce bacterial infection (through the use of AMP) and promote cell proliferation and migration, enhance collagen deposition and angiogenesis, thereby effectively promoting rapid healing of infected wounds. The synthesis of the precursor materials was then analyzed by Fourier transform infrared spectroscopy (FTIR) and nuclear magnetic resonance spectroscopy ^1^H-NMR. The physical properties of the dressing were also characterized by water absorption, water vapor permeability and porosity studies. In addition, the wound dressings were evaluated by *in vitro* antimicrobial assay, *in vitro* degradation, cellular assay and animal assay for their antimicrobial capacity, biodegradability, biocompatibility and ability to promote healing of infected wounds.

## 2 Materials and methods

### 2.1 Materials

Polyvinyl alcohol (PVA, CAS: 9002-89-5, alcoholysis: 97.5–99 mol%, viscosity: 3.5–4.5 mPa⋅s) and N,N-diethylethylenediamine (DEEDA, CAS: 111-74-0) were purchased from Shanghai Aladdin Biochemistry Technology Co., Ltd. (Shanghai, China). Hyaluronic acid (HA, CAS: 9067-32-7, molecular weight: 150,000-250,000) was obtained from Shanghai Yuanye Bio-Technology Co., Ltd. (Shanghai, China). Antimicrobial peptide (AMP, KRWWKWWRRC, purity>95%) was obtained from Shanghai Apeptide Co., Ltd. (Shanghai, China). Tetrakis (4-bromomethylphenyl) ethylene (TPE, CAS: 67448-47-9, purity: 90%) was purchased from Zhengzhou Alpha Chemical Co., Ltd. (Zhengzhou, China). Sodium alginate (ALG, CAS: 9005-38-3, viscosity: 350–550 mPa⋅s^-1^), N,N′-Carbonyldiimidazole (CDI, CAS:530-62-1, purity: 99%), N-(3-Dimethylaminopropyl)-N′-ethylcarbodiimide hydrochloride (EDC, CAS: 25952-53-8, purity: 98.5%) and N-hydroxysuccinimide (NHS, CAS: 6066-82-6, purity: 98%) were obtained from Shanghai Macklin Biochemical Co., Ltd. (Shanghai, China). Silkworm cocoon was obtained from Chinese Academy of Agricultural Sciences. All other chemical reagents were analytically pure and purchased from Shanghai Aladdin Biochemistry Technology Co., Ltd. (Shanghai, China).

### 2.2 Synthesis of PVA-TPE

We synthesized PVA-TPE with reference to previous studies, and the synthesis was carried out in steps (Gao et al., 2016). First, the PVA modification (synthetic PVA-DEEDA) was carried out. Briefly, a quantity of PVA (10.0 g) was weighed into a 500 mL round bottom flask, 200 mL of DMSO was added and heated to 90°C to dissolve it completely. After complete dissolution, when the temperature of the solution had dropped to 25°C, an appropriate amount of CDI (18.4 g) was added to activate the above solution for 3 h. When the reaction was complete, 16 mL of DEEDA was then slowly added to the above solution for 24 h at room temperature. To remove unreacted CDI, 100 mL of ammonia solution was added to the final solution and the reaction was stirred for 1.5 h. Finally, the mixture was precipitated by adding 10 times acetone and the precipitate was dissolved in a suitable amount of deionized water for dialysis. The dialyzed solution was lyophilized to obtain modified PVA (PVA-DEEDA).

This was followed by the synthesis of PVA-TPE. The PVA-DEEDA synthesized in the above steps was weighed and dissolved in DMSO, heated and stirred at 80°C to dissolve. TPE dissolved in DMSO was slowly added dropwise to the above solution, and the mixture was further stirred at 70°C for 24 h. Finally, appropriate amount of acetone was added for precipitation, the product was dissolved in pure water and dialysed, and freeze-dried to obtain pure PVA - TPE.

### 2.3 Synthesis of HA-AMP

HA-AMP was synthesized according to the literature with some modifications (Wei et al., 2021). An aqueous HA solution of 4 mg/mL was prepared, then AMP powder (500 mg) was added to the aqueous HA solution and stirred magnetically for about 4 h to obtain a clear solution. EDC (750 mg) and 1-hydroxybenzotriazole (660 mg) were then dissolved in 10 mL of DMSO/H_2_O (1:1) solution and slowly added to the above clarified solution. The pH of the mixed solution was adjusted to 4.75 and the reaction was carried out for 4 h, then the pH was adjusted to 7.0 to complete the reaction. At the end of the reaction, the mixed solution was dialyzed (MWCO 8–15 kDa). The final HA-AMP powder was obtained by lyophilization.

### 2.4 Preparation of silk fibroin (SF)

SF was prepared as described above (Zhu et al., 2022). Silkworm cocoons (10 g) were degummed in 2 L Na_2_CO_3_ (0.5 mM) solution at 100°C for 30 min, washed several times with distilled water and the above procedure was repeated three times. After degumming, the silk protein was dried in an oven to obtain the degummed silk protein. Dissolve 10 g of the above degummed filipin protein in 80 mL of lithium bromide (9.3 M) solution and add 0.48 g of NaOH to react for 1 h at 60°C, then add 1 mL of HCl to neutralize the unreacted NaOH and continue the reaction for 3 h. At the end of the reaction, the solution was filtered to remove insoluble matter and then transferred to a dialysis bag (MW: 12,000-14,000 Da) and dialyzed for 48 h with distilled water. The solution was dialyzed for 48 h. The solution was lyophilized and collected to obtain filipin protein (SF).

### 2.5 Preparation of wound dressings

SF (3%), ALG (3%) and HA-AMP (1%) were dissolved in deionized water to form homogeneous aqueous solutions. The above aqueous solutions were mixed in 48-well plates according to the volume ratios of ALG: SF: HA-AMP = 1:1:1, 1:2:1 and 1:1:2, lyophilized and then added with ethanol solution of EDC and NHS to cross-link for 48 h. After cross-linking, the plates were rinsed several times with gradient concentration ethanol solutions to remove residual EDC, NHS, after lyophilization, different ratios of SF/ALG/HA-AMP dressings were obtained and named as ALG/SF/HA-AMP (1/1/1), ALG/SF/HA-AMP (1/2/1), and ALG/SF/HA-AMP (1/1/2), respectively.

In addition, an aqueous solution of HA (1%) was prepared, and the concentrations of other solutions were referred to the above method. The solution was mixed according to the volume ratio of ALG: SF: HA-AMP = 1:1:1, and then PVA-TPE (3 mg/mL) was added and fully dissolved in 48-well plates, which were lyophilized and then added with EDC and NHS ethanol solution to cross-link for 48 h. After cross-linking, the wound dressings were washed several times with gradient concentrations of ethanol solution to remove residual EDC and NHS, and then lyophilized to obtain SF/ALG/HA-AMP/PVA-TPE wound dressings. Meanwhile, ALG/SF/HA and SF/ALG/HA/PVA-TPE wound dressings were prepared as above.

### 2.6 Chemical characterization of synthetic materials

To determine the structure of HA, HA-AMP, PVA, PVA-TPE, the samples were dissolved using a nuclear magnetic resonance (^1^H-NMR) spectrometer (Inova-600M, Varian, United States) with deuterated heavy water as a solvent, placed in a nuclear magnetic tube for testing and analysis, and graphically analyzed using MestReNova software. In order to analyze the changes in the functional groups of HA, HA-AMP, PVA and PVA-TPE, the samples were thoroughly ground with an appropriate amount of potassium bromide powder (approximately 5% by mass) using a Fourier Transform Infrared (FTIR) spectrometer (VERTEX70, Bruker, Germany), pressed (vacuum pressure of 20 mmHg, pressing time of 5 min) and analyzed in the wavelength range of 4,000–500 cm^-1^.

### 2.7 Scanning electron microscopy (SEM) analysis

The lyophilized wound dressings were demolded and their surfaces were sprayed with gold for 30 s. The surface morphology was observed using a scanning electron microscope (S-3400 Hitachi, Japan) at an accelerating voltage of 5 kV.

### 2.8 Porosity

The porosity of wound dressings is also one of the main factors affecting their properties, which we characterised by the ethanol solution displacement method (Wang et al., 2017; Liu et al., 2018). The method was as follows: a known volume (V_1_) of ethanol was poured into a graduated container containing the sample, taking care that the ethanol submerged the sample. The container was placed in a desiccator and vacuumed for 1 h. The sample was sufficiently moistened to remove all the air from the pores of the sample and the volume at this point was recorded as V_2_. After removing the sample, the volume of the remaining ethanol was recorded as V_3_. The porosity was calculated according to the formula:

$$\text{Porosity }\left(\%\right)=\left[\left(V_{1}-V_{3}\right)\;/\;\left(V_{2}-V_{3}\right)\right]\times 100\;\%$$ (1)

### 2.9 Water absorption

The absorbency properties of the dressings were assessed using the specific gravity method (Ma et al., 2019; Yuan et al., 2020). The pre-weighed dressing stent (m_0_) was placed in a certain volume of PBS buffer solution (pH = 7.4) and immersed for a set period of time at 37°C, then the saturated dressing stent was taken out and weighed (m_t_). The water absorption was calculated as:

$$\text{Water absorption }\left(\%\right)=\left[\left(m_{t}-m_{0}\right)\;/\;m_{0}\right]\times 100\;\%$$ (2)

### 2.10 Water vapour transmission rate (WVTR)

The pre-weighed sample to be tested (W_0_) was clamped in a permeable cup and the bottle area of the permeable cup was measured (s) (Deng et al., 2023). The samples were incubated at 37°C for a specified time (t), the water vapor permeability was measured using the difference in humidity between the two sides of the sample, and the final mass of the samples was weighed (W_1_) at the end of the incubation. The following formula was used to calculate the water vapour transmission rate (WVTR):

$$\text{WVTR }\left(g/{\text{cm}}^{2}\;*\;24\;h\right)=\left(W_{0}-W_{1}\right)\times 24)/\left(s\times t\right)\;$$ (3)

### 2.11 Tensile properties

The material to be tested was cut into a rectangle of 75 × 15 mm and its thickness (d) was measured using a thickness gauge at room temperature and 60% humidity, then the tensile mechanical properties were determined using a universal materials testing machine and the load at failure of the specimen was recorded (F) (Chen H. et al., 2023). The following parameters were determined: tensile speed of 10 mm/min, clamping distance of 65 mm and specimen width of 15 mm (b). The tensile strength (Ób) was calculated using the formula:

$$Ób\;\left(\text{MPa}\right)=\left[F\;/\;\left(b\times d\right)\right]$$ (4)

Simultaneously, the elongation at break of the material is tested. The original length of the sample to be tested is recorded as L and the displacement value of the specimen at break after stretching by the universal testing machine is recorded as L'. Then the elongation at break (E) of the specimen is calculated as:

$$E\;\left(\%\right)=\left[\left(L^{\prime }-L\right)\;/\;L\right]\times 100\;\%$$ (5)

### 2.12 Degradation performance

The *in vitro* degradability of a dressing can be assessed by its mass loss (Chen Z. et al., 2023). The dressings were freeze-dried and weighed to determine the mass, recorded as W_1_, and then the original dressings were immersed in a solution of PBS or lysozyme (1000 U/mL) and incubated at 37°C, 70 rpm. For measurement, the dressing was removed for washing and its lyophilised mass was recorded as W_2_. The *in vitro* degradation rate (D) of the samples was calculated using the formula:

$$D\;\left(\%\right)=\left[\left(W_{1}-W_{2}\right)\;/\;W_{1}\right]\times 100\;\%$$ (6)

### 2.13 Fluorescence spectrum

Aqueous solutions of SF/ALG/HA-AMP/PVA-TPE with a masterbatch concentration of 1,000 μg/mL were configured according to a concentration gradient of 1,000, 800, 600, 400, 200, 100, 50, 25, 10, and 0 μg/mL. Fluorescence spectra were measured at an excitation wavelength of 360 nm and an emission wavelength of 200–800 nm using a fluorescence spectrophotometer (UV 1800, China).

### 2.14 Evaluation of cytocompatibility

*Evaluation of cell viability* (Deng et al., 2023)*:* wound dressings were sterilised overnight by UV irradiation, added to complete DMEM medium at a mass ratio of 1:10, macerated for 24 h, filtered through a 0.22 μm sterile filter head and set aside. 3T3 cells were cultured in 48-well plates to make adherence (5,000/well). After cell attachment, the medium was aspirated and subsequently added to the wound dressing extract for 24 h. After incubation, the extract was aspirated, washed with PBS and incubated for 1–2 h (37°C, 5% CO_2_) with 500 μL/well of CCK-8 working solution (DMEM basal medium containing 10% CCK-8). After incubation, the absorbance (OD) was measured with an enzyme marker at 450 nm and the cell survival rate (CSR) was calculated according to the formula:

$$\text{CSR }\left(\%\right)=\left[\;\left({\text{OD}}_{\text{sample}}-{\text{OD}}_{\text{blank}}\right)/\left({\text{OD}}_{\text{control}}-{\text{OD}}_{\text{blank}}\right)\;\right]\times 100\;\%$$ (7)

*Live-dead cell staining* (Deng et al., 2023)*:* To further assess cell survival, the LIVE/DEAD Cell Imaging Kit was used to stain 3T3 cells. For live/dead cell staining, after aspirating and discarding the medium, PBS was rinsed, and then live/dead cell staining working solution (2 µM calcein AM, 8 µM PI) was added and incubated (25°C, 20 min); washed once with PBS; and immediately after adding the anti-fluorescence quencher, the images were captured with a fluorescence microscope to analyse cell survival.

### 2.15 Evaluation of *in vitro* antimicrobial properties

*Escherichia coli* (*E. coli*) and *S. aureus* were resuscitated and cultured to logarithmic growth stage centrifuged and resuspended (1 × 10^8^ CFU/mL). The bacterial suspension and wound dressing were co-incubated at 37°C for 4 h. Gradient dilutions were applied to LB agar plates and counted after 12 h of incubation (Tamer et al., 2016). Besides, the bacteria co-cultured with the material were collected, centrifuged (5,000 rpm, 10 min), resuspended, and stained by avoiding light (3 μL, Syto-9: PI = 1:1, 15 min), and the excess dye was removed by washing with PBS. Photographs were taken for observation by confocal laser microscopy (CLSM) (Hassan et al., 2022; Lin et al., 2023). Meanwhile, bacteria co-cultured with the material were collected and fixed in 1 mL of 2.5% glutaraldehyde for 2–3 h after multiple centrifugations (5,000 rpm, 20 min). Then it was cleaned by centrifugation several times, dispersed in deionized water, and 10 μL drops were taken on monocrystalline silicon wafers, dried naturally, sprayed with gold for 90–120 s, and photographed.

### 2.16 Mouse model of infected wounds

The mouse wound infection model was modelled on previous studies with minor modifications (Sun et al., 2022; Wang et al., 2023). Mice (male, C57BL/6) of 25 ± 5 g were selected and anaesthetized by intraperitoneal injection of 0.5% pentobarbital sodium (45–60 mg/kg). After anesthesia, the mice were removed from the back and surrounding hair with an animal shaver and depilated with depilatory cream, followed by disinfection of the exposed skin with 75% alcohol. The dorsal skin was gently lifted at 5 mm on either side of the midline of the back, and circular wounds with a diameter of 10 mm were cut, and 10 μL of a mixture of *E. coli* and *S. aureus* at 1 × 10^8^ CFU/mL was added dropwise to each wound, and the experiments were performed 1 day after infection. The infected mice were randomly divided into four groups: blank control group, HA/SF/ALG dressing group, HA-AMP/SF/ALG dressing group, and HA-AMP/SF/ALG/PVA-TPE dressing group. The wound was closed with different materials according to the above grouping, and then covered with 3 M Tegaderm waterproof adhesive tape on the outer layer for fixation, and the wound healing was observed and the wound closure rate was calculated at 3, 7, 10, and 14 days after the operation, during which the medical sterile gauze or hydrogel dressing was not changed. Meanwhile, the wound tissue was taken and placed in PBS for ultrasonication to obtain bacterial suspension, which was applied to the plate to observe the antimicrobial status of the wound dressing (Sun et al., 2022). In addition, the skin and tissues around the wounds of mice on 3, 7, 10 and 14 days were collected for subsequent experiments.

### 2.17 Histological analysis and immunohistochemical evaluation

The skin and tissues collected in step *2.17* were fixed in 4% paraformaldehyde for more than 24 h, and then subjected to subsequent steps such as dehydration, embedding, slicing, and staining (H&E and Masson staining) (Zhu et al., 2022). At the same time, according to the standard procedure, immunohistochemical staining was performed with antibodies of VEGF (abcam, ab1316), TGF-β1 (proteintech, 21898-1-AP), TNF-α (abcam, ab6671), IFN-γ (proteintech, 15365-1-AP) and IL-4 (proteintech, 66142-1-Ig).

### 2.18 Western blot analysis

Mouse skin tissue proteins were analysed qualitatively and semi-quantitatively using standard Western blotting methods. We used the following antibodies: GAPDH (proteintech, 60004-1-Ig, 1: 1000), WNT5A/B (proteintech, 55184-1-AP, 1: 1000), CAMK2 (proteintech, 13730-1-AP, 1: 1000), PKC (Servicebio, GB11650, 1: 1000) and P-PKC (ABclonal, AP-1045, 1: 1000).

### 2.19 Statistical analysis of data

All data were statistically analysed using the software Origin and GraphPadPrism 8. Data are shown as mean ± standard deviation. Significant differences in data: *p* * <0.05, *p* ** <0.01and *p* *** <0.001.

## 3 Results and discussions

### 3.1 Characterization of the chemical composition of dressings and observation of microscopic morphology

**FIGURE 1:**
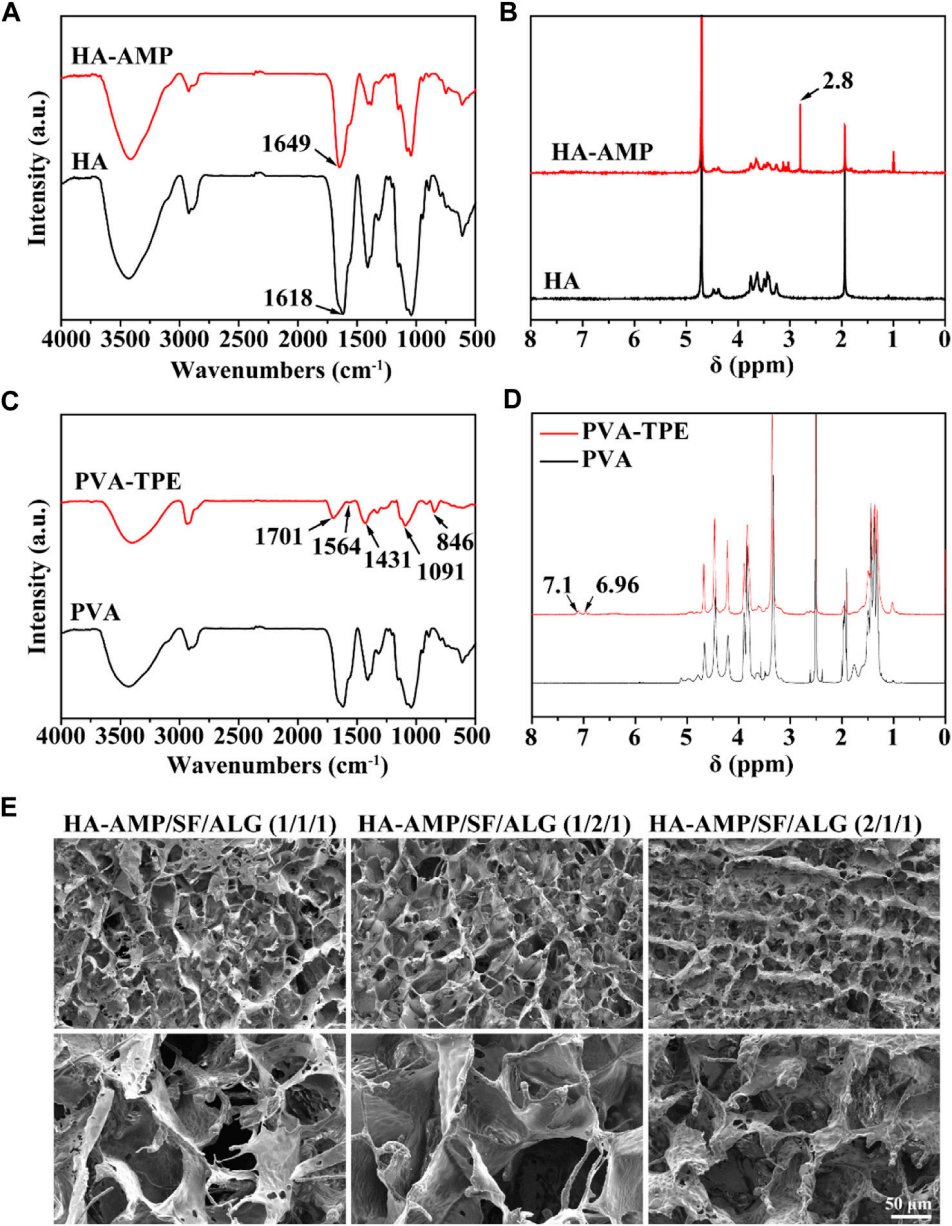
Chemical structure of dressing preparation materials and microscopic morphology of the dressings. **(A)** FTIR spectra of HA and HA-AMP; **(B)** NMR hydrogen spectra of HA and HA-AMP; **(C)** FTIR spectra of PVA and PVA-TPE; **(D)** NMR hydrogen spectra of PVA and PVA-TPE; **(E)** micro-morphology of three composition ratios of HA-AMP/SF/HA dressings taken by SEM.

FT-IR and ^1^H-NMR were used to verify the synthesis of HA-AMP and PVA-TPE. From Figure 1A, it can be seen that HA grafted AMP showed a strong characteristic absorption peak of sec-amide (-NH) at 1,643 cm^–1^, while -NH_2_ in HA belonging to the hyaluronic acid chain was 1,620 cm^–1^, which was due to the condensation of the carboxyl group on the activated HA with the amino group on the AMP. Meanwhile, a new 1,380 cm^–1^ attributed to -CH_3_ existed on HA-AMP, indicating that the synthesis of HA-AMP was successful. HA-AMP is a copolymer formed by grafting AMP onto the HA backbone based on the EDC/NHS coupling technique, in which the carboxyl group on HA is activated by EDC/NHS and the carboxyl group undergoes a condensation reaction with the amino group on AMP (Wei et al., 2021). NMR hydrogen spectra showed a peak of ester group at 2.8 ppm position, indicating the successful preparation of HA-AMP (Figure 1B). The functional group changes of PVA-TPE are shown in Figure 1C, and there is a strong characteristic absorption peak of -C=O in the infrared spectrum (1701 cm^–1^), which is formed after the quaternisation reaction of PVA with DEEDA. At the same time, the introduction of the triethylamine group (1,091 cm^–1^) contained on DEEDA on PVA indicated the success of PVA chain modification; while 1,564, 1,431, and 846 cm^–1^ were the characteristic absorption peaks of 1,4-substituted benzene ring, indicating the successful grafting of TPE on PVA. The appearance of the 7.0 ppm peak in the 1H-NMR spectrum in Figure 1D indicated that the TPE unit was successfully grafted (Wang et al., 2020).

The morphology of the dressings was observed by SEM (Figure 1E), and all the dressings had a three-dimensional connected mesh structure with uniform pore size distribution (Song et al., 2023). Obviously, we observed a slight difference in the pore size of the dressings with different volume ratios, due to the fact that the increase in the volume ratio of SF or HA-AMP increases the intermolecular interaction force, which slightly increases the pore size of the dressings, but the change is not significant. Too large a pore size will make the scaffold too loose, and too fast water absorption will easily cause wound infiltration, so the appropriate pore size is more conducive to cell adhesion and growth to promote wound healing (Fernández-Pérez and Ahearne, 2019; Wei et al., 2021).

### 3.2 Physical properties of dressings and fluorescence luminescence of PVA-TPE

**FIGURE 2:**
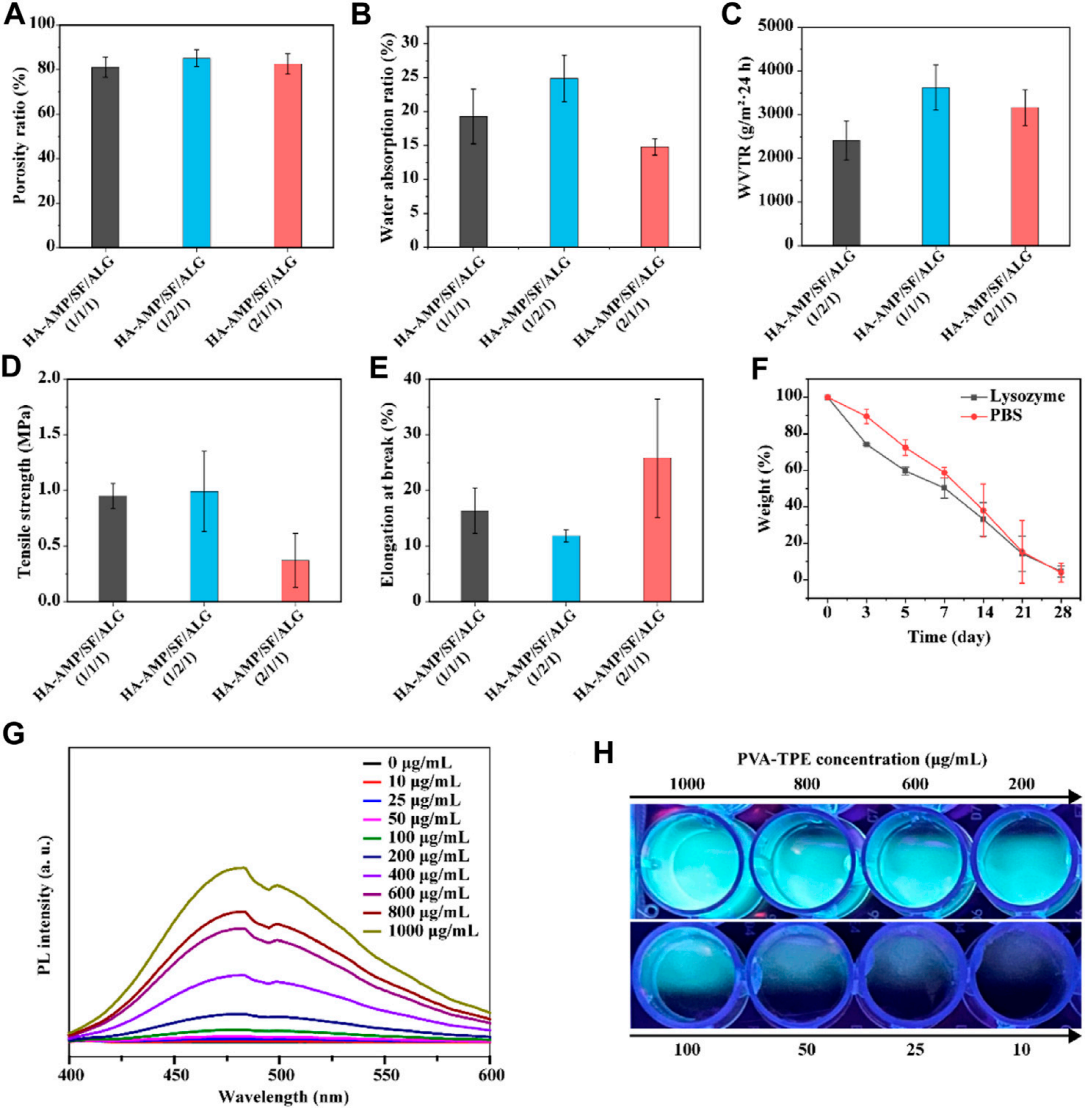
Physical properties and fluorescence luminescence of the dressings. **(A)** Porosity, **(B)** water absorption, **(C)** water vapor transmission rate, **(D)** tensile strength and **(E)** elongation at break of HA-AMP/SF/ALG dressings with different composition ratios; **(F)** *in vitro* degradation of HA-AMP/SF/ALG (1/1/1) dressings in a PBS environment and a lysozyme environment; **(G)** fluorescence emission spectra of different concentrations of PVA-TPE curves and **(H)** fluorescence images under UV illumination.

It can be seen from Figure 2A that the porosity of different samples was 81.13% ± 4.50%, 85.14% ± 3.73%, 82.55% ± 4.54%. With the increase of HA-AMP or SF, the porosity of the dressing sample increased. The explanation of this phenomenon is that with the increase of the volume of HA-AMP or SF solution, the intermolecular force is enhanced, so that the pores are increasing during crosslinking, which eventually leads to the gradual increase of the pores of the wound dressing sample formed by drying, resulting in an increase in porosity. As shown in Figure 2B, the water absorption rates were 19.29% ± 4.02%, 24.86% ± 3.40%, and 14.81% ± 1.22%, which was attributed to the SF molecule contains a large number of hydrophilic functional groups (e.g., carboxyl and hydroxyl), which could enhance the hydrophilicity and water retention of the scaffolds to improve connectivity and hydrophilicity of the porous scaffolds and to promote the migration and cell proliferation (Zhao X. et al., 2016). While a larger water absorption rate is prone to rapid dehydration of the wound tissue, leading to the formation of larger scar tissue at the wound surface, a smaller water absorption rate is prone to the aggregation of tissue osmotic fluid, which plays a negative role in wound healing. Figure 2C shows the WVTR at different ratios, and they show significant differences. The use of a dressing with a higher WVTR value on the wound surface may trigger rapid dehydration of the wound tissue and formation of scar tissues at the wound surface; whereas, a lower WVTR will impede the exchange of gases between the wound tissue and the external environment, which may result in the anaerobic bacterial propagation and accumulation of tissue exudate, which may lead to wound inflammation or even ulceration (Zhang D. et al., 2015; Wang et al., 2017). Therefore, the dressing with a volume ratio of 1:1:1 was chosen as the optimal group for subsequent experiments. The reason for the other two groups to be larger may be the presence of silk protein or hyaluronic acid, which leads to the presence of larger gaps in the dressing after cross-linking, resulting in an increase in the WVTR value.

The biodegradability of a good wound dressing should match the growth process of the injured tissue (He et al., 2021). The degradation properties of the wound dressings varied with time. The degradation rates represented by both curves showed a decreasing trend with increasing immersion time. In comparison, the dressings showed a faster degradation rate in the lysozyme solution. Therefore, the degradation of this material is governed by the external environment, with biological enzymes acting as a mediating environment to accelerate the hydrolysis and dissolution of biomaterials (Ma et al., 2019) (Figure 2F).

The results of testing the tensile strength (Figure 2D) and elongation at break (Figure 2E) of the dressings showed that the higher the content of HA-AMP, the higher the elongation at break of the dressings, but correspondingly lower the tensile strength. Taken together, the tensile strength and elongation at break of HA-AMP/SF/ALG dressings were in the moderate range. In order to verify the fluorescence intensity of polymer PVA-TPE after grafting on TPE molecules, we tested the fluorescence intensity of PVA-TPE at different concentrations, respectively. As shown in Figure 2G, with the enhancement of concentration, the hydrophobic TPE units gradually aggregated, which led to a gradual increase in the intensity of the emission peak at 475 nm (Zhao Y. et al., 2018; Wang et al., 2018). A clear fluorescence luminescence could be seen under UV illumination when the concentration of PVA-TPE was 100 μg/mL (Figure 2H).

### 3.3 Assessment of biocompatibility of wound dressings

**FIGURE 3:**
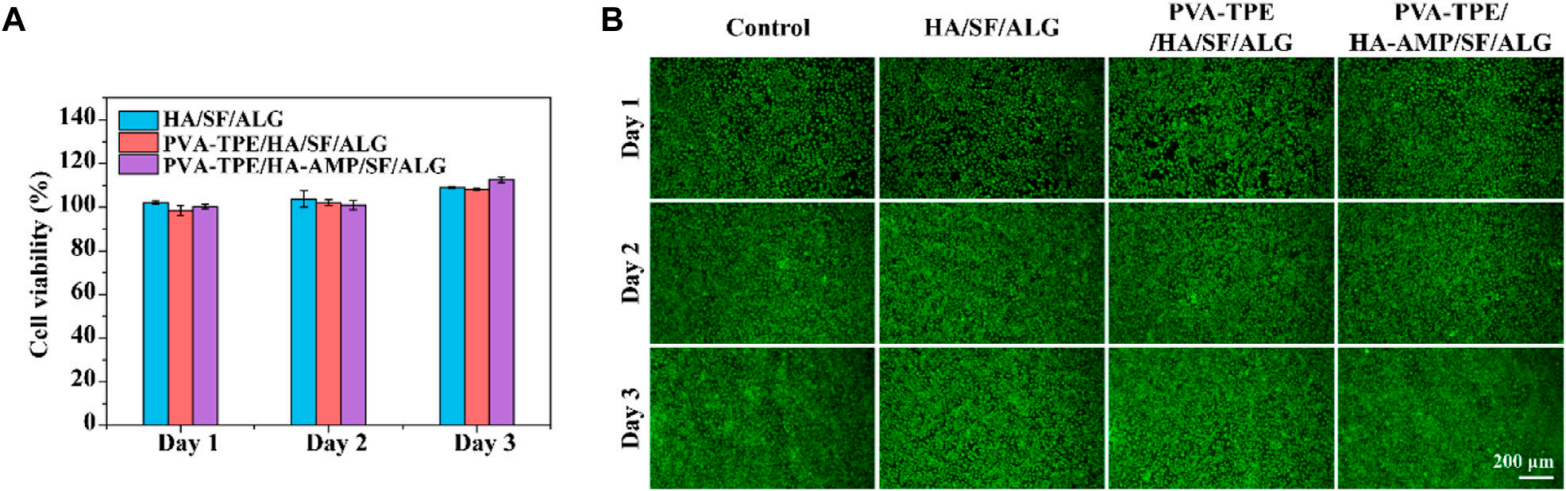
Cytotoxicity and cell migration ability of dressings. **(A)** Cell viability determined by CCK-8 assay after co-culture of dressing extract with 3T3 cells; **(B)** staining of live and dead 3T3 cells.

Biomedical materials are directly used in the human body or are closely related to human health, so in addition to the mechanical properties required for general application materials, the biocompatibility evaluation of materials is also essential in the research design of biomedical materials (Raguvaran et al., 2017). Therefore, following the test protocol in ISO 16886.5, we used wound dressing extracts co-cultured with 3T3 (mouse embryonic fibroblasts) and evaluated their biocompatibility by detecting cell viability and cell viability staining (Lin et al., 2019). The results are shown in Figure 3A, after quantification of cell viability by CCK-8 method, cell survival was observed to be greater than 100% within 1–3 days, proving that our prepared dressing was not cytotoxic. Green fluorescence in Figure 3B represents live cells and red fluorescence represents dead cells. From the figure, it can be observed that the cells co-cultured with the dressing extract had good viability, the number of dead cells was very small, and basically no difference with the control group was observed. In addition, fibroblasts are one of the main cells in the wound healing process, secreting growth factors and collagen fibers to promote wound closure (Gan et al., 2019). Therefore, the dressing we prepared has the potential to accelerate wound healing.

### 3.4 Evaluation of the *in vitro* antimicrobial activity of wound dressings

**FIGURE 4:**
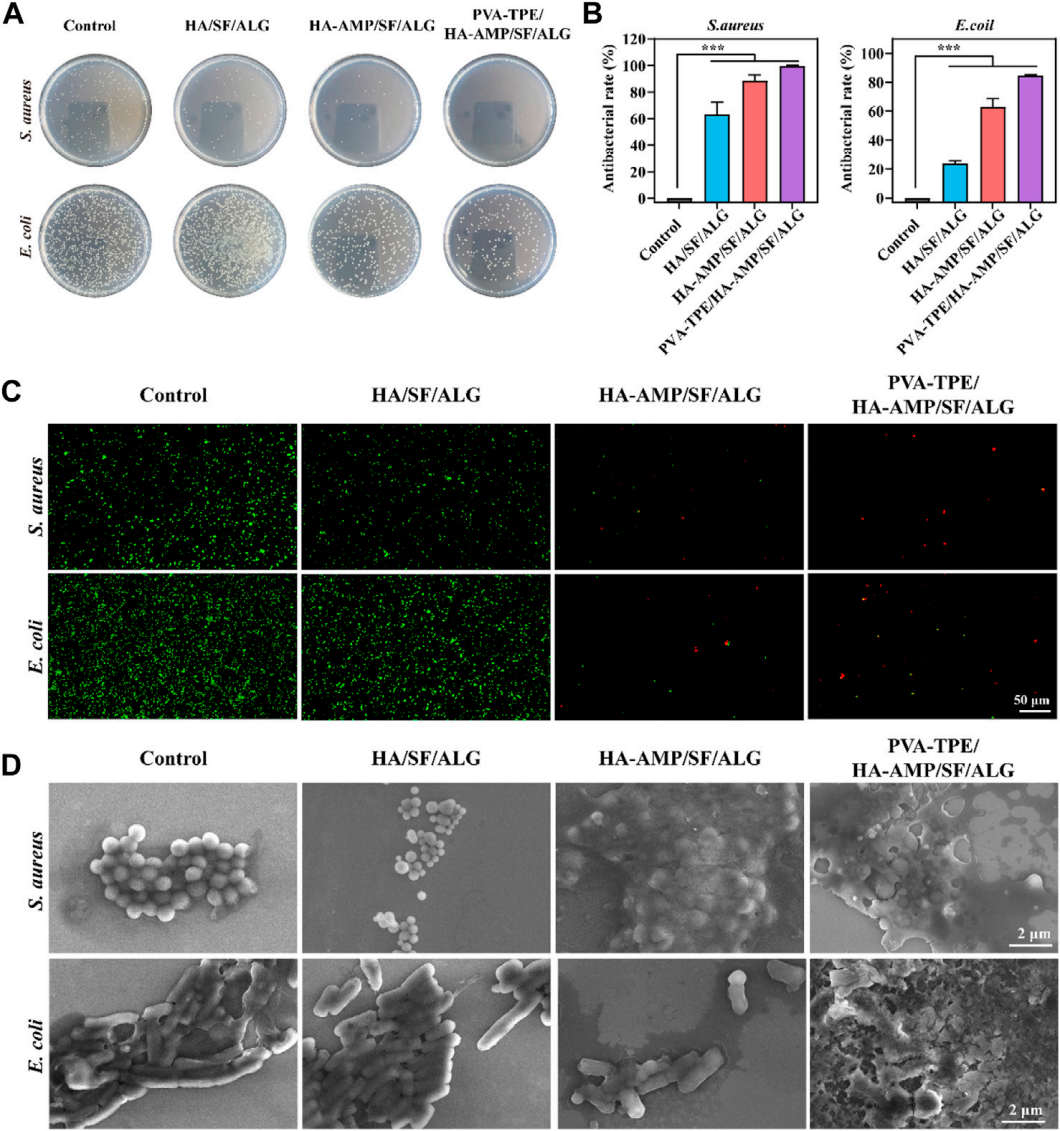
*In vitro* antimicrobial evaluation of dressings. **(A)** Distribution of colonies on agar plates after co-culturing with dressings and dilution of bacterial fluids; **(B)** statistics of antimicrobial resistance of dressings against *E. coli* and *Staphylococcus aureus*; **(C)** live-dead fluorescence staining images of *E. coli* and *Staphylococcus aureus* after co-cultivation with different dressings and **(D)** SEM images of bacterial morphology. ***p < 0.001.

Facing the threat of bacterial infection, antimicrobial activity has become a basic requirement for wound dressings. Here, the antimicrobial properties of wound dressings were investigated using *E. coli* and *S. aureus* as models, and the results are shown in Figure 4. The growth of colonies on agar plates after incubation with the dressings is shown in Figure 4A. The PVA-TPE/HA-AMP/SF/ALG dressings showed the strongest antimicrobial effect with the lowest number of colonies, followed by the HA-AMP/SF/ALG dressings with AMP. The statistics of the antibacterial rate results in Figure 4B showed that the PVA-TPE/HA-AMP/SF/ALG dressing showed an antibacterial rate of 99.99% against *S. aureus* and 84.63% against *E. coli*. Subsequently, live-dead fluorescence staining was performed on the survival status of bacteria, and the results are shown in Figure 4C. Red fluorescence represents dead bacteria. Both *E. coli* and *S. aureus* cultured on PVA-TPE/HA-AMP/SF/ALG dressing showed obvious death. After observing the bacterial morphology by scanning electron microscopy (Figure 4D), we found that both PVA-TPE/HA-AMP/SF/ALG dressing and HA-AMP/SF/ALG cultured bacteria had some rupture or change in morphology, and the bacteria were encapsulated by ruptured plasma. Especially, the morphology of the bacteria treated with PVA-TPE/HA-AMP/SF/ALG dressing was almost completely changed, and it was difficult to observe morphologically intact bacteria. This suggested that the introduction of HA-AMP and PVA-TPE can improve the antimicrobial effect of wound dressings, increased their antibacterial activity on wounds, and inhibited the growth of bacteria well. The possible reasons are: a) the antimicrobial peptide is able to interfere with the synthesis of the bacterial cell wall and exerts an antimicrobial effect, and b) PVA-TPE is a TPE molecule grafted *in situ* by quaternisation with the properties of AIE, which combines both quaternary ammonium groups and AIE groups. The quaternary ammonium compounds have a bactericidal effect when the positively charged quaternary ammonium portion binds to the negatively charged bacterial cell membrane under electrostatic interactions.

### 3.5 Evaluation of healing of infected wounds in mice

**FIGURE 5:**
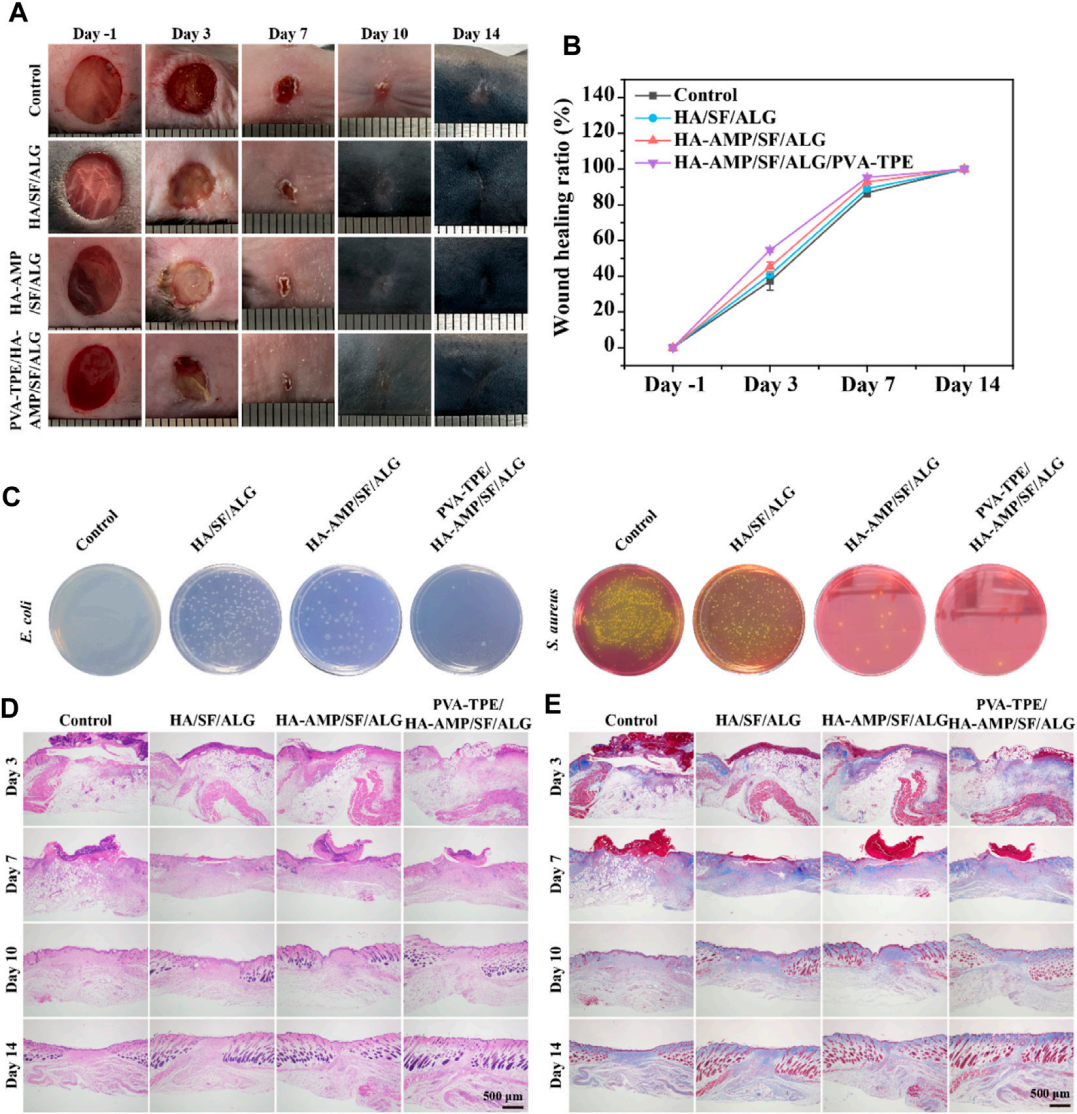
Evaluation of healing of infected wounds. **(A)** Mouse wound healing macrograph and **(B)** wound healing rate; **(C)** images of *in vivo* wound bacterial colonies on selective medium agar plates; **(D)** H&E staining and **(E)** Masson staining of skin at the wound site.

Although it has been verified from *in vitro* experiments that the wound dressing has excellent *in vitro* antimicrobial capacity, we further verified its therapeutic effect on infected wounds from the *in vivo* treatment route. By taking photos and analyzing the wound conditions at different time points, we can see that a bacterial film appeared on the surface of all wounds on day 0 (2 days after bacterial infection), indicating that they were all infected. After 10 days of treatment, the dressing had a faster rate of wound closure and was able to completely close the wounds. The mice treated with HA-AMP/SF/ALG/PVA-TPE dressing had the fastest rate of healing, with the wounds healing significantly faster than the other two groups and the presence of a yellowish bacterial film was no longer observed (Figures 5A, B). The above results show that HA-AMP/SF/ALG/PVA-TPE wound dressing has a significant enhancement effect on tissue regeneration, confirming its positive repairing effect on wounds. This may be attributed to its continuous AMP delivery, which sustained effective inhibition of bacterial growth, which contributed to wound healing and excellent biocompatibility (Lin et al., 2019).

To assess the *in vivo* antimicrobial properties of the wound dressings, after taking the skin on the third day for bacterial quantification, it was found that HA-AMP/SF/ALG dressing was able to significantly reduce the bacterial counts, which indicated that it had strong antimicrobial capacity. The combined treatment of AMP and PVA-TPE in HA-AMP/SF/ALG/PVA-TPE dressing resulted in the best antimicrobial effect with the least number of colonies (Figure 5C). This indicates that very effective antimicrobial activity can be obtained after applying the wound dressing to infected wounds.

Severely infected wounds are usually accompanied by a strong inflammatory response. Here, we used H&E staining to assess inflammatory cells and granulation formation in the repaired tissues after 3, 7, 10 and 14 days of treatment with the dressings (Figure 5D). The re-epithelialization of the HA-AMP/SF/ALG/PVA-TPE dressing and HA-AMP/SF/ALG dressing was faster than that of the remaining two groups, and a more intact epidermal layer could be basically observed on the seventh day. These results confirmed that our newly designed HA-AMP/SF/ALG/PVA-TPE dressing has a positive restorative ability, which not only reduced infection but also promoted granulation tissue deposition by releasing AMP and TPE. This suggested that removing bacterial infection from wounds is significant in accelerating wound healing, which meets our need for controlling bacterial infection in casualties and accelerating wound closure in battlefield environments (Prasad, 2013; Zhang L. J. et al., 2015).

Collagen is another important indicator for assessing dermal formation during wound healing. We used Masson trichrome staining to analyze collagen formation during the later stages of wound healing (Figure 5E). The collagen deposition shown by Masson staining indicated that the group treated with HA-AMP/SF/ALG/PVA-TPE dressing did not show a significant abnormal increase in collagen, reducing the possibility of scarring. This phenomenon suggests that delivery of AMP and TPE during wound healing may be more effective in improving collagen deposition, reconfirming the positive restorative effects of HA-AMP/SF/ALG/PVA-TPE dressings.

### 3.6 Angiogenesis and inflammatory response in wound healing

**FIGURE 6:**
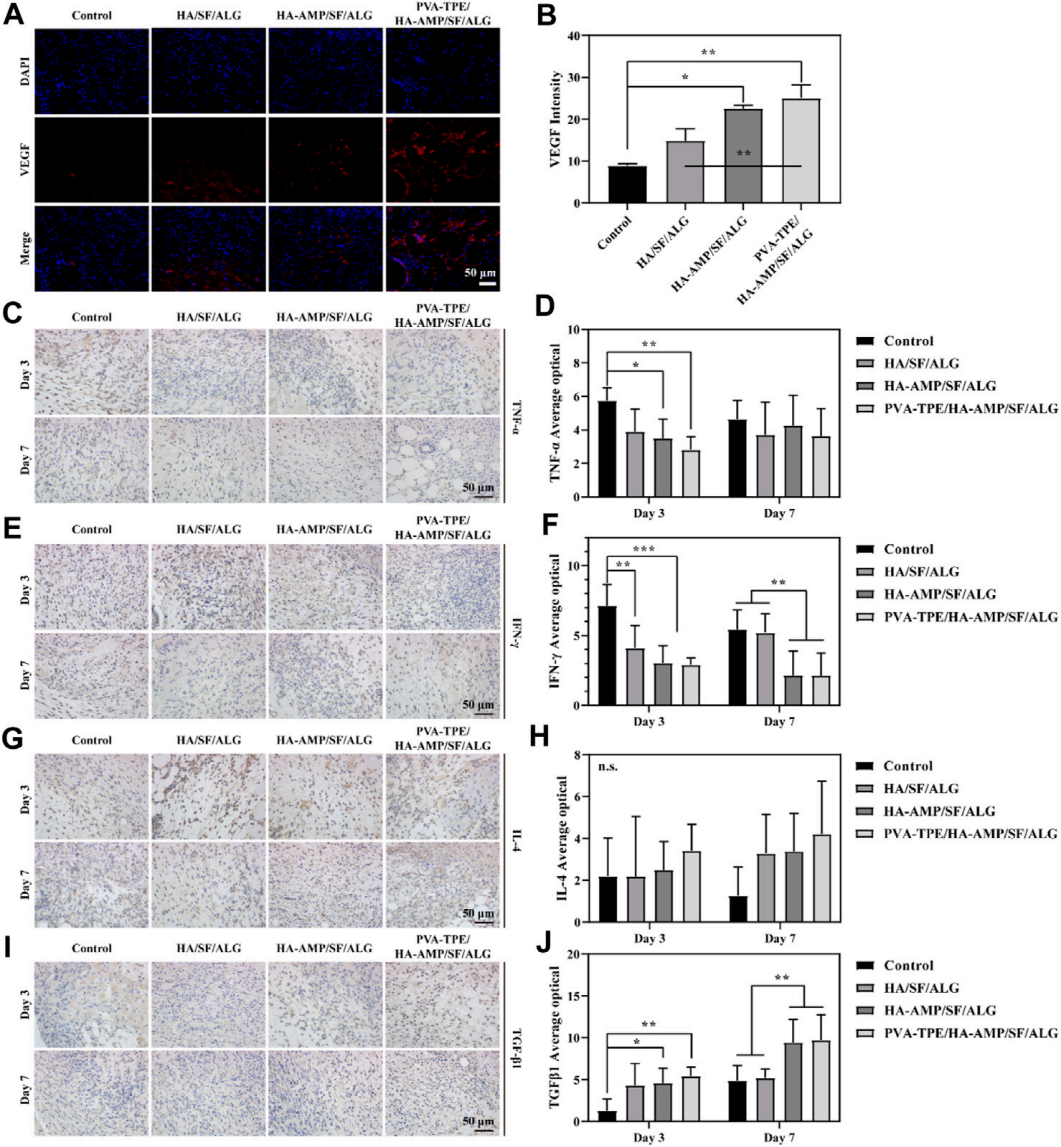
Expression levels of VEGF and inflammatory factors at the trauma site. **(A)** Immunofluorescence staining images of VEGF and **(B)** intensity statistics of VEGF expression; **(C)** Immunohistochemical staining images of TNF-α expression at the injury site after 3 and 7 days of trauma treatment and **(D)** quantitative statistics of positivity rate; **(E)** Immunohistochemical staining images of IFN-γ expression at the injury site after 3 and 7 days of trauma treatment and **(F)** quantitative statistics of positivity rate; **(G)** Immunohistochemical staining images of IL-4 expression at the injury site after 3 and 7 days of treatment and **(H)** quantitative statistics of positivity rate; **(I)** Immunohistochemical staining images of TGF-β1 expression at the injury site after 3 and 7 days of treatment and **(J)** quantitative statistics of positivity rate. *p < 0.05, **p < 0.01, ***p < 0.001.

Vascular endothelial growth factor (VEGF) signaling plays a key role in blood vessel formation and growth, stimulating angiogenesis, promoting endothelial cell proliferation and migration, and increasing vascular permeability (Zhang et al., 2023). Compared with the control group, all VEGF intensities in the wound dressing group increased to some extent. Most obviously, the HA-AMP/SF/ALG/PVA-TPE dressing had the greatest VEGF intensity (Figures 6A, B), which indicated that the combined treatment of AMP and TPE in the dressing further upregulated VEGF expression, which was beneficial to promote blood vessel formation and growth.

As an important inflammatory factor, tumor necrosis factor-α (TNF-α) can produce different effects on different types of cells. In addition, TNF-α is also an important immune regulatory molecule, which plays an important role in many inflammatory and autoimmune diseases (Müller et al., 1987). Immunohistochemical staining of TNF-α protein in mouse wound tissue and its relative expression was counted after 3 and 7 days of treatment. Compared with the control group, the relative expression of TNF-α proteins within the group treated with wound dressings were reduced to some extent. Among them, the HA-AMP/SF/ALG/PVA-TPE dressing group had the lowest relative expression of TNF-α protein and the best effect, which indicated that it could inhibit the occurrence of inflammatory reaction and shorten the healing time of the wound (Figures 6C, D).

Interferon γ (IFN-γ) is a tight anti-inflammatory indicator, a highly active multifunctional secretory protein, and a small molecule polypeptide that regulates cellular immune function. As the most dominant macrophage-stimulating factor in the body, IFN-γ activates macrophages to perform phagocytosis and clear pathogens (Rožman and Švajger, 2018). The immunohistochemical staining and quantitative statistics of positivity for the expression of IFN-γ (Figures 6E, F) showed similar results to TNF-α, suggesting that our dressing can stimulate macrophages to function and participate in the inflammatory response.

Interleukin 4 (IL-4) plays an important role in the treatment and research of tumours and autoimmune diseases, in addition to its role in mediating inflammatory allergic responses. It enhances intercellular interactions and can induce cells to divide, proliferate and differentiate (Le Moine et al., 1999). After 3 days of treatment of infected wounds of mice with wound dressing, the statistics of IL-4 expression in wound tissue showed that the intensity of IL-4 expression was in the following order: HA-AMP/SF/ALG/PVA-TPE > HA-AMP/SF/ALG > HA/SF/ALG. After 7 days of treatment, the intensity of IL-4 expression was further increased in the following order (Figures 6G, H). It indicated that our wound dressing could promote cell proliferation and differentiation for wound healing, and AMP and TPE could further promote this effect.

Transforming growth factor β1 (TGF-β1) can be involved in proliferative scar formation by regulating inflammatory response with fibroblast growth and metabolism, and is currently recognised as an important indicator associated with pathological scarring (Guo et al., 2017; Taylor et al., 2017). The results of the study showed that the treatment group using the dressing was significantly able to increase TGF-β1 expression. After 7 days of treatment, TGF-β1 expression was more significantly upregulated in the HA-AMP/SF/ALG/PVA-TPE *versus* HA-AMP/SF/ALG dressing groups, suggesting that they were able to induce wound self-repair and reduce scar formation (Figures 6I, J).

### 3.7 Dressing promotes wound healing by enhancing the expression of Wnt/CAMK/p-PKC signalling pathway

**FIGURE 7:**
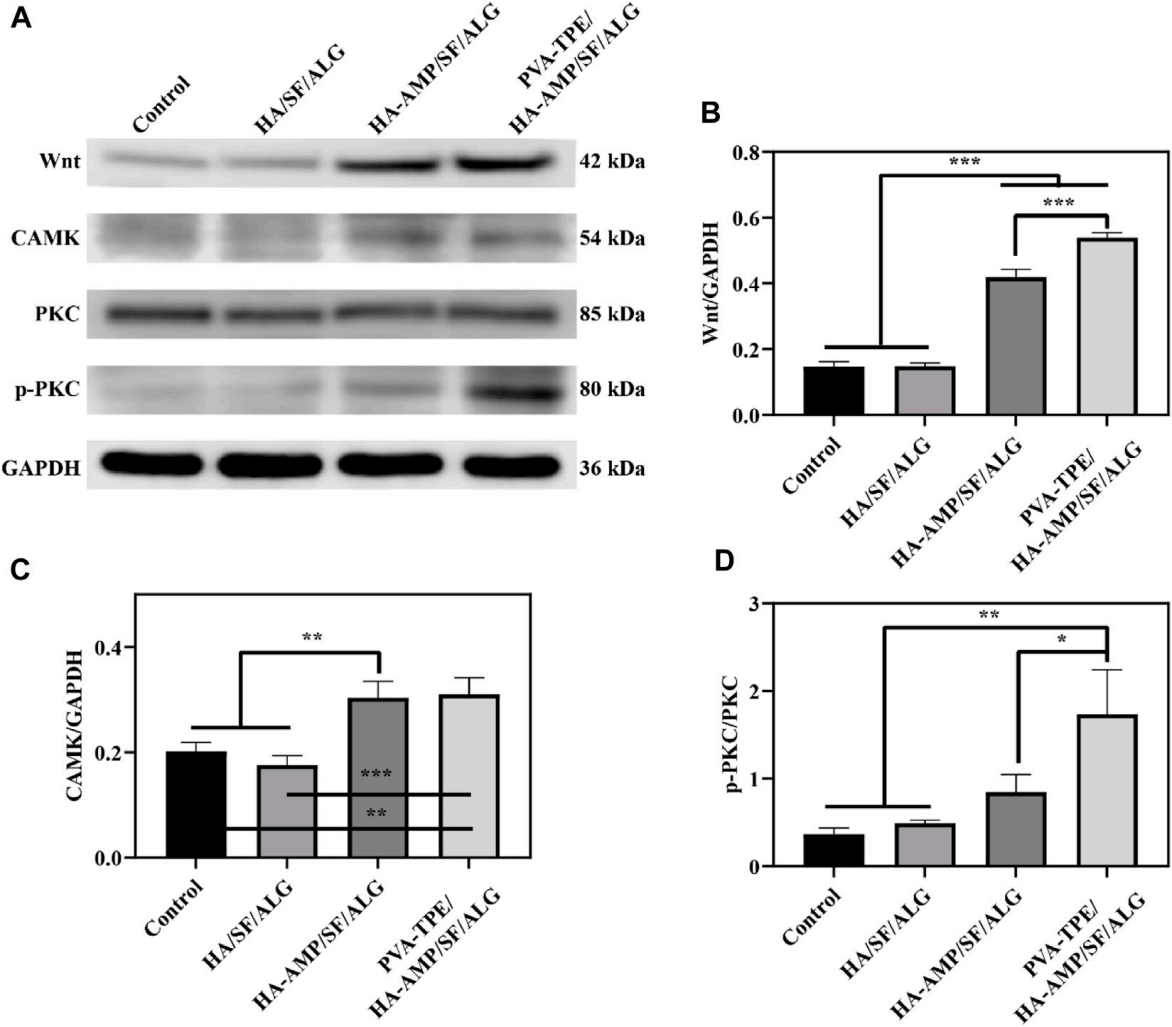
Quantitative statistics of WB bands and grey values of healing-related signalling pathway proteins after 7 days of treatment. **(A)** WB bands of Wnt, CAMK, PKC and p-PKC; **(B)** relative expression of Wnt, **(C)** CAMK and **(D)** p-PKC. *p < 0.05, **p < 0.01, ***p < 0.001.

Wound healing is a complex, dynamic process regulated by multiple cell signalling pathways (Ren et al., 2015; Teufel and Hartmann, 2019). The Wnt signalling pathway is a set of signal transduction pathways with multiple downstream channels stimulated by the binding of the ligand protein Wnt and membrane protein receptors. As one of the signaling pathways involved in skin healing, Wnt signaling pathway is considered to have many functions such as regulating the development of skin and its appendages, inducing the morphogenesis of skin appendages, regulating the periodic growth of hair follicles, promoting wound angiogenesis and epithelial remodeling (Nie et al., 2020). Calcium/calmodulin-dependent kinase II (CaMK II) is an important class of regulatory proteins that can regulate the biological behaviour of cells through modifications such as autophosphorylation and oxidation (Gu et al., 2017). Protein kinase C (PKC) is an important class of molecules that regulate the biological behaviour of cells (cell growth, differentiation, metabolism and transcription). After taking skin samples on day 7 for pathway analysis and quantitative analysis of tissue healing related signals, we found that PVA-TPE/HA-AMP/SF/ALG wound dressing may enhance cell proliferation and promote wound neovascularization by up-regulating the expression of Wnt and CAMK signaling pathways (Figures 7A–C). Also, by up-regulating the expression of p-PKC, which in turn regulates cell proliferation and reduces inflammatory response (Figure 7D).

### 3.8 *In vivo* biosafety evaluation

**FIGURE 8:**
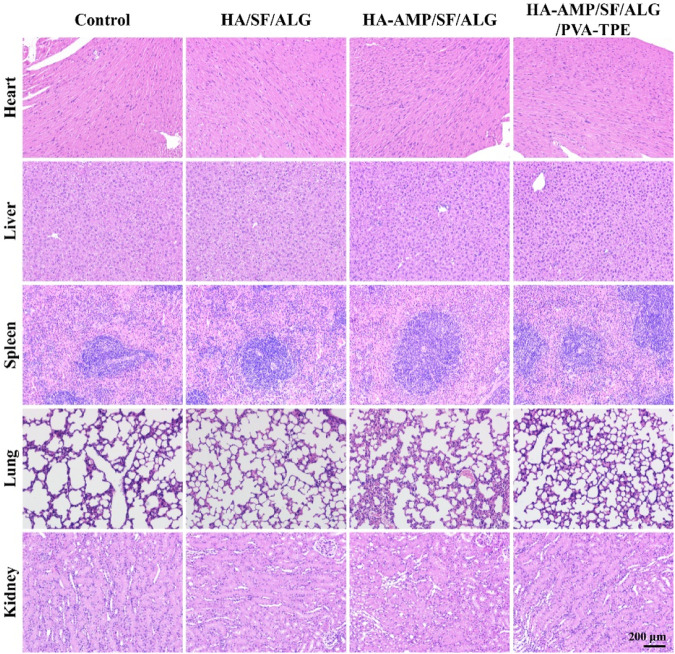
H&E staining of major organs.

H&E staining of major organs was performed after completion of treatment to observe the *in vivo* biosafety of the dressings. By comparing the morphology of heart, liver, spleen, lungs and kidneys through the results of tissue sections of each group (Figure 8), we saw that the dressing group (especially PVA-TPE/HA-AMP/SF/ALG wound dressing) showed higher epidermal layer intactness, which indicated faster reconstruction of damaged wound tissue (Teng et al., 2021). Meanwhile, the morphology of heart, liver, spleen, lungs and kidneys was normal, proving that our prepared dressings are not toxic and have the prospect of battlefield transformation.

## 4 Study limitations

Composite wound dressings have shown great potential for application in the healing of infected wounds in rats, but there are some limitations, such as: a) Whether wound dressings are able to act on pre-traumatic haemorrhage needs to be further evaluated. b) The long term biosafety of wound dressings needs to be fully explored in small or large mammals. c) The potential of composite wound dressings in the healing of infected wounds in rats needs to be fully explored in small or large mammals.

## 5 Conclusion

In this study, PVA-TPE/HA-AMP/SF/ALG wound dressing smart response system by EDC/NHS chemical crosslinking method. It endows the hydrogel with excellent antimicrobial properties through the slow release of AMP. Meanwhile, the addition of PVA-TPE enables the system to monitor bacterial activity and guide precise antimicrobial activity. Firstly, we characterized the physicochemical properties of this novel wound dressing smart response system, which not only proved that it has suitable porosity, excellent water absorption and water vapor permeability, but also excellent mechanical properties and *in vitro* degradation ability. Secondly, through cellular and bacterial experiments, we demonstrated that the wound dressing has excellent biocompatibility, the ability to promote cell proliferation and migration, and also has excellent antibacterial properties. Finally, we applied the wound dressing to infected wounds in mice to further evaluate its ability to repair wounds from *in vivo*. It was finally demonstrated that the PVA-TPE/HA-AMP/SF/ALG wound dressing could promote the healing of infected wounds. Meanwhile, it could induce the expression of inflammatory factors such as VEGF, TNF-α, IFN-γ, IL-4 and TGF-β1 in infected wounds through the Wnt/CAMK/p-PKC signaling pathway, which could inhibit inflammatory responses, promote wound healing and reduce scar formation. Therefore, the PVA-TPE/HA-AMP/SF/ALG wound dressing smart response system may offer a promising strategy for patients with wound infections.

## Data Availability

The original contributions presented in the study are included in the article/Supplementary material, further inquiries can be directed to the corresponding authors.
